# More doctors: better attention to the population’s health?^*^

**DOI:** 10.1590/S1516-31802012000300001

**Published:** 2012-07-12

**Authors:** José Luiz Gomes do Amaral, Paulo Manuel Pêgo-Fernandes, Benoit Jacques Bibas

**Affiliations:** I Titular Professor of the Discipline of Anesthesiology, Pain and Intensive Care, Escola Paulista de Medicina, Universidade Federal de São Paulo; President of the World Medical Association.; II Associate Professor of the Discipline of Thoracic Surgery, Hospital das Clínicas, Faculdade de Medicina da Universidade de São Paulo; Scientific Director of Associação Paulista de Medicina.; III Trainee Physician in Tracheal Surgery and Therapeutic Respiratory Endoscopy, Discipline of Thoracic Surgery, Hospital das Clínicas, Faculdade de Medicina da Universidade de São Paulo.

## THERE IS NO LACK OF PHYSICIANS IN BRAZIL, OR ANY EVIDENCE THAT THEIR NUMBERS WILL BECOME INSUFFICIENT OVER THE NEXT 20 YEARS

Over the last decade, the number of physicians in Brazil grew by 21.3%, a rate that was greater than the increase in the country’s population over the same period (12.3%).^1^ Today, more than 350,000 physicians are registered in Brazil.^1^ Taking into account that the country’s population has surpassed 190 million,^2^ there are now 1.8 physicians per 1,000 inhabitants. Thus, each physician has 544 people to care for.

Brazilian physicians can be attributed the same life expectancy as the population that they attend, i.e. 73 years. They will probably work until they reach the age of 70 years. Therefore, it can be supposed that, from the time of gaining specialist certification until retirement, they will have 40 years of professional life. It is legitimate to estimate that 1 in 40, or 8,750, will leave the profession every year. On the other hand, there are 181 medical schools,^3^ from which 90% of the students admitted graduate, which means 13,500 new physicians every year.^1^ Out of the 350,000 physicians active today, 87,500 will have left the profession by 2021, but 135,000 new physicians will have been added to those who remain.

The Brazilian Institute for Geography and Statistics (Instituto Brasileiro de Geografia e Estatística, IBGE) has estimated that the country’s population will be 208,280,241 inhabitants in 2021 and 216,410,030 inhabitants in 2030.^2^ In 2021, there will be 397,500 physicians, i.e. 524 patients per physician or 1.9 physicians per 1,000 inhabitants. In 2030, there will be 445,000 physicians, i.e. 485 patients per physicians or 2.0 physicians per 1,000 inhabitants.

There are insufficient resources in the Brazilian healthcare system (both public and private) to maintain the existing numbers of physicians.

In 2010, a survey published by the Getúlio Vargas Foundation (Fundação Getúlio Vargas) showed that the average weekly workload of physicians with postgraduate qualifications was 52 hours and their monthly remuneration was R$ 8,966.07.^4^ Thus, taking into consideration that labor-law burdens will be added to this salary of R$ 8,966.07, the employer’s mean annual expenditure on this physician will be R$ 198,150.14.

Thus, the existing physicians’ employment contracts represent an investment of the order of R$ 70 billion per year, which is equivalent to almost the entirety of the Ministry of Health’s budget.^5^ Without taking into consideration the limited investments that Brazil makes in its chaotic healthcare system, this suggests that the solution for the system would be to move Brazil towards the European figures of 2.5 to 3 physicians per 1,000 inhabitants. This fallacious argument is convenient because it would justify increasing the number of places in Brazilian medical schools by 2,500. Would it be possible to hire the services of so many physicians? If a ratio of 2.5 physicians per 1,000 inhabitants is taken, there would need to be 833,120 physicians for the Brazilian population of 2021 (208,280,241 inhabitants); there would need to be 865,640 physicians for the population expected in 2030 (216,410,030 inhabitants). Hiring all of these physicians would cost R$ 165 billion in 2021 and R$ 171.5 billion in 2030.

## THE DISTRIBUTION OF PHYSICIANS IN BRAZIL REFLECTS THE COUNTRY’S SOCIAL AND ECONOMIC DEVELOPMENT AND ILLUSTRATES THE PREDOMINANCE OF INVESTMENT IN THE PRIVATE HEALTHCARE SYSTEM

The number of physicians in Brazil is not the target that should be corrected but, rather, the distortions in their distribution. Seventy-two percent of the physicians in Brazil are in the states of the southern and southeastern regions: not coincidentally, these are the regions where the private healthcare system has the greatest participation.^1^ The Federal District leads the ranking, with 4.02 physicians per 1,000 inhabitants, followed by Rio de Janeiro (3.57), São Paulo (2.58) and Rio Grande do Sul (2.31), with rates comparable to those of European countries.^1^ At the other end of the scale are Amapá, Pará and Maranhão, with less than one physician per 1,000 inhabitants.^1^ This serious problem results from the absolute lack of consistent policies and public resources for taking medicine into the non-metropolitan interior of the country.

Opening new medical courses will not resolve the attendance deficiencies, but poor-quality medical schools tend to worsen these problems. Seventy-seven medical schools were created between 2000 and 2010: 7.7 per year or more than one every 60 days! This is equivalent to 42.5% of the schools opened over two centuries in Brazil.^1^ Doubling the number of medical schools over this period has not solved the poor distribution of physicians, and lack of attendance has continued, including in some major urban centers.

## IN MANY OF THE EXISTING MEDICAL SCHOOLS, THE TEACHING IS OF VERY POOR QUALITY

Indiscriminate opening of medical schools is a very highly relevant issue. The lack of seriousness in dealing with this matter has resulted in the current somber situation. A recent assessment by the Regional Medical Council of the State of São Paulo (Conselho Regional de Medicina do Estado de São Paulo) among sixth-year students attested that almost 50% of them did not know how to interpret radiographs or make simple diagnoses.^6^ They would administer inappropriate treatment for common conditions such as tonsillitis, meningitis and syphilis, and would be incapable of identifying fever as a risk factor for serious infections in neonates.^6^

If half of the 13,500 new graduates from medical schools are incapable of getting 50% of diagnoses or prescriptions right, and if they attend 10 patients a day, on five days a week, for 48 weeks a year, they will make 8,100,000 mistakes every year (33,750 per day; 168,750 per week; 8,100,000 per year; and 324 million over the course of 40 years of their professional lives).

In a situation in which there are no resources to maintain so many physicians, it is going to be even less possible to find a way of paying for so many mistakes.

As usual, within a situation in which impunity is the rule among those in power, the healthcare and education authorities have evaded their responsibilities and the bill will, once again, be paid by physicians and their patients.
